# Complete genome sequence of *Helicobacter pylori* isolated from residents in southwestern Colombia using Oxford Nanopore sequencing technology

**DOI:** 10.1128/mra.00082-25

**Published:** 2025-05-20

**Authors:** Lizeth Mejia, Julie Benavides-Melo, Ernesto Argoty, Liliana Montenegro, Nelson Rivera-Franco, Diana López-Alvarez, Álvaro Pazos

**Affiliations:** 1 Laboratorio de Técnicas y Análisis Ómicos-TAOLab/CiBioFi, Facultad de Ciencias Naturales y Exactas, Universidad del Valle 28006, Cali, Valle del Cauca, Colombia; 2 Departamento de Biología, Facultad de Ciencias Exactas y Naturales, Universidad de Nariño 27994, Pasto, Nariño, Colombia; 3 Grupo GIISE, Facultad de Medicina, Universidad Cooperativa de Colombia, Pasto, Nariño, Colombia; 4 Alcaldía de Pasto, Secretaria de Salud–Salud Pública, Pasto, Nariño, Colombia; 5 Grupo HOSDERNAR, Hospital Universitario Departamental de Nariño 173055 , Pasto, Nariño, Colombia; 6 Grupo VIREM‐Virus Emergentes y Enfermedad, Escuela de Ciencias Básicas, Facultad de Salud, Universidad del Valle, Cali, Valle del Cauca, Colombia; 7 Departamento de Ciencias Biológicas, Facultad de Ciencias Agropecuarias, Universidad Nacional de Colombia 140920, Palmira, Valle del Cauca, Colombia; 8 Grupo de investigación Salud Pública, Centro de Estudios en Salud, Universidad de Nariño (CESUN), Pasto, Nariño, Colombia; University of Maryland School of Medicine, Baltimore, Maryland, USA

**Keywords:** whole-genome sequencing, *Helicobacter pylori*, precancerous gastric lesions

## Abstract

Genome sequences of *Helicobacter pylori* strains isolated from patients in Nariño, Colombia, with gastric lesions were assembled using Nanopore sequencing. Plasmids were detected in some strains and were predicted to be mobilizable, with relaxases of the MOBP type. *H. pylori*’s virulence genes may explain the link with gastric cancer.

## ANNOUNCEMENT

*Helicobacter pylori*, a pathogen colonizing the gastric mucosa of over half the global population (1), induces gastric inflammation, though most infected individuals remain asymptomatic. However, a significant percentage develop gastric or duodenal ulcers (10%–15%) or gastric cancer (1%–3%) (2).

This study analyzed gastric biopsies from the residents of high- and low-risk gastric cancer regions in Nariño, Colombia. Histopathological diagnosis and culturing of antral biopsies were performed according to established protocols (3). Histopathology revealed non-atrophic gastritis (six patients), atrophic gastritis with intestinal metaplasia (one patient), and diffuse gastric cancer (one patient) (Table 1). *H. pylori* strains were identified by Gram staining and urease, oxidase, and catalase tests. The gastric biopsy sample was cultured on Columbia agar (Oxoid, UK) supplemented with 10% sheep defibrinated blood, selective supplement Dent (Oxoid, UK), and 1% enrichment supplement Isovitalex (Oxoid, UK), under 10% CO_2_ at 37°C for 7–10 days, then cryopreserved in NUNC tubes with 80% sterile thioglycolate and 20% glycerol at −80°C. For DNA extraction, bacterial cultures were grown under the same conditions, but without Dent, using the UltraClean Blood DNA Isolation Kit (MOBIO) and quantified with a Qubit version 3.0 fluorometer (Thermo Fisher Scientific).

**TABLE 1:** Assembly genomic statistics and gene predictions and annotations of Colombian *H. pylori* strains using Nanopore sequencing^*a*^

Strain ID	GenBank accessions	Diagnosis	Age	Sex	Host residence	SRA accession	Raw reads	Reads *N*_50_	Genome size (bp)	Mean coverage (×)	No. of contigs	Contig *N*_50_ (bp)	CDS (total)	rRNAs	tRNAs	ncRNAs	Lineage
CR12	CP175941, CP175942	NAG	33	F	Florida	SRR26717568	809,317	536	1,706,154	193.6	2	1,700,838	1,601	6	36	3	hspAfrica1LatinAmerica
CR41	JBLDXM000000000	NAG	30	F	Barbacoas	SRR26717567	225,971	477	1,612,862	49.9	31	108,384	1,596	4	36	3	hspAfrica1LatinAmerica
CR44	CP175940	AGIM	36	M	Pasto	SRR26717566	1,192,402	2,111	1,681,553	787.4	1	1,681,553	1,586	6	36	3	hspSWEuropeLatinAmerica
CR45	JBLDXL000000000	NAG	34	M	Pasto	SRR26717565	1,635,951	453	1,734,444	345.7	70	44,824	1,683	6	38	3	hspSWEuropeLatinAmerica
CR46	CP175939	NAG	45	M	Samaniego	SRR26717564	1,842,573	840	1,623,746	653.4	1	1,623,746	1,528	6	36	3	hspSWEurope
CR56	JBLDXK000000000	DGC	24	M	Pasto	SRR26717563	282,596	600	1,712,951	78.8	36	111,822	1,669	4	37	3	hspSWEuropeLatinAmerica
CR60	CP175938	NAG	47	F	Pasto	SRR26717562	3,332,538	833	1,672,799	1,129.5	1	1,672,799	1,561	6	36	3	hspSWEuropeLatinAmerica
CR71	JBLDXJ000000000	NAG	34	F	Pasto	SRR26717561	803,778	403	1,683,646	151.1	31	132,000	1.652	5	36	3	hspSWEuropeLatinAmerica

^*a*^ F, female; M, masculine; NAG, non-atrophic gastritis; AGIM, atrophic gastritis with intestinal metaplasia; and DGC, diffuse gastric cancer.

MinION sequencing libraries were prepared with ligation sequencing kit (SQK-LSK109) and native barcoding expansion kit (SQK-NBD104) (Oxford Nanopore Technologies, ONT). No DNA shearing or size selection was performed before library preparation. Sequencing was performed on a flow cell (R9.4.1) for 72 h on the MinION (ONT). Default parameters were used for all software unless otherwise specified. Fast5 reads were converted to fastq with Guppy version 6.5.7 in high-accuracy mode (4). Raws fastq were trimmed by cutadapt version 4.9 (5) according to the needs of each sample. The reads were assembled using Flye version 2.9.4 (6) and polished with Pilon version 1.24 (7) (--fix “bases,” “gaps”), Medaka version 2.0.1 (ONT, 2018), and homopolish version 0.4.1 (8). Genome completeness was assessed with BUSCO version 5.5.0 (9) and quality with checkM version 1.2.2 (10). Circularity was determined using Flye version 2.9.4 (6), and coverage depth with Qualimap version 2.2.2 (11). Gene predictions and annotations were performed using PGAP (12). The assembled genomes showed an average GC content of 38%, a size of 1.7 Mbp, and 1,593 protein-coding genes (Table 1).

Four plasmids were detected using MOB-Suite version 3.1.0 (13) in strains CR12 (CP175942), CR41 (JBLDXM010000005 and JBLDXM010000021), and CR71 (JBLDXJ010000026). Plasmids ranged in size from 4,260 to 9,178 bp, with three plasmids classified as mobilizable and containing MOBP-type relaxases (AB508, AE952, and novel plasmid from CR71).

**Fig 1:**
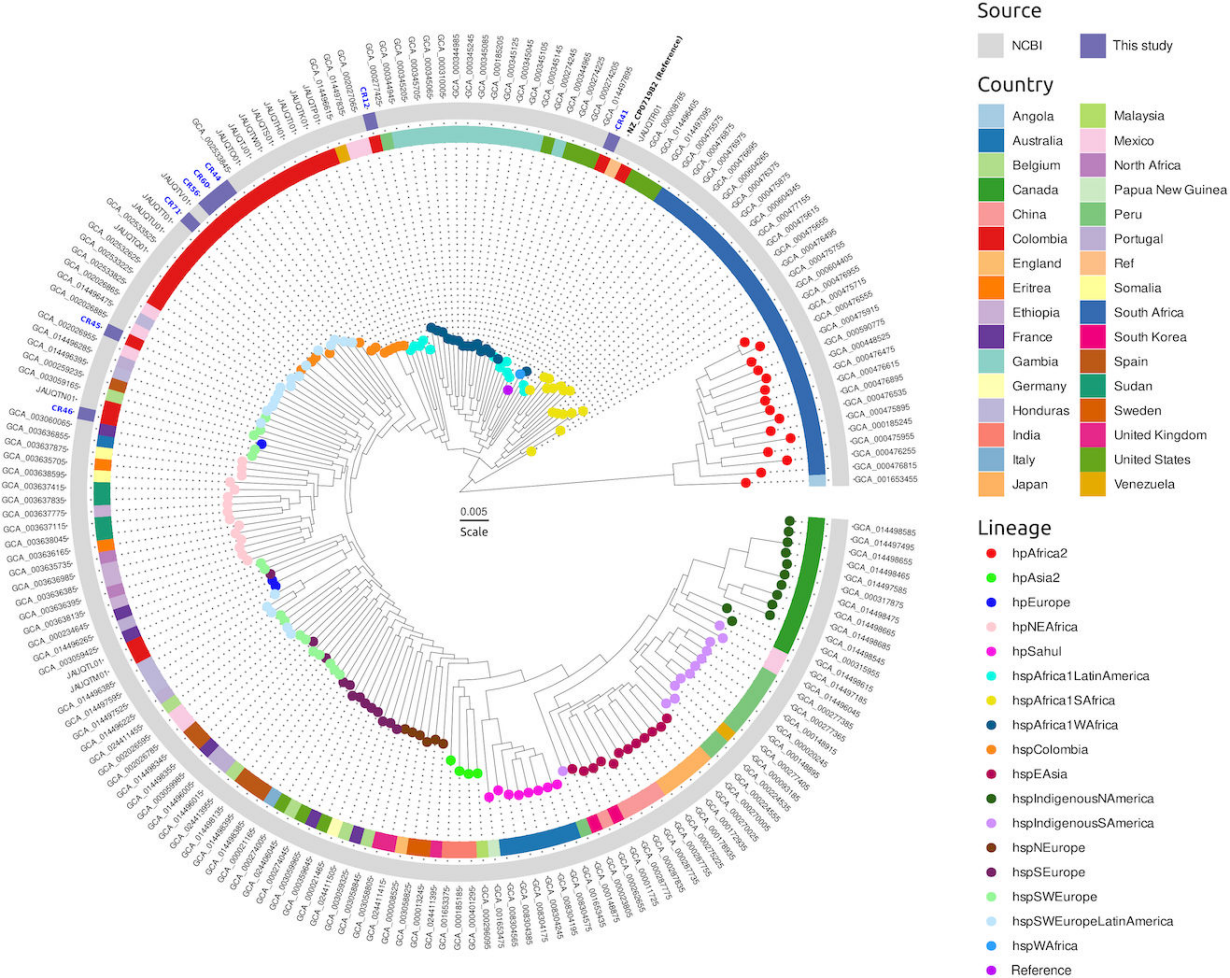
Maximum likelihood tree of the full database (192 sequences) of genomes of *H. pylori*. Color annotations are given in the circles around the terminal nodes, indicating the country of origin for the *H. pylori* isolates included in the tree. The color of the tips represents the lineage from which each genome was reported. The gray area indicates NCBI genomes, and the blue one the genomes obtained in this study.

Phylogenetic analysis, based on SNP alignments generated with RealPhy version 1.12, was performed with IQ-TREE version 2.2.6 (14) and visualized with ggtree version 3.12.0 (15) and ggtreeExtra version 1.14.0 (16) packages. A total of 184 *H*. *pylori* genomes from NCBI (PRJNA529500) were included, representing previously defined genetic populations and subpopulations (17, 18). The analysis identified four genomes related to hspSWEuropeLatinAmerica, two to hspAfrica1LatinAmerica, one to hspSWEurope, and one to hspColombia (Fig. 1). Raw FASTQ and assembled genomes are available in NCBI under BioProject PRJNA1037030 (Table 1).
